# Artificial Intelligence and Antibiotic Discovery

**DOI:** 10.3390/antibiotics10111376

**Published:** 2021-11-10

**Authors:** Liliana David, Anca Monica Brata, Cristina Mogosan, Cristina Pop, Zoltan Czako, Lucian Muresan, Abdulrahman Ismaiel, Dinu Iuliu Dumitrascu, Daniel Corneliu Leucuta, Mihaela Fadygas Stanculete, Irina Iaru, Stefan Lucian Popa

**Affiliations:** 1 2nd Medical Department, “Iuliu Hatieganu” University of Medicine and Pharmacy, 400000 Cluj-Napoca, Romania; lilidavid2007@yahoo.com (L.D.); abdulrahman.ismaiel@yahoo.com (A.I.); popa.stefan@umfcluj.ro (S.L.P.); 2 Faculty of Environmental Protection, University of Oradea, 410048 Oradea, Romania; 3 Department of Pharmacology, Physiology and Pathophysiology, Faculty of Pharmacy, “Iuliu Hațieganu” University of Medicine and Pharmacy, 400000 Cluj-Napoca, Romania; cmogosan@umfcluj.ro (C.M.); cristina.pop.farmacologie@gmail.com (C.P.); cazacuirina16@gmail.com (I.I.); 4 Department of Computer Science, Technical University of Cluj-Napoca, 400027 Cluj-Napoca, Romania; zoltan.czako@cs.utcluj.ro; 5 Department of Cardiology, “Emile Muller” Hospital, 68200 Mulhouse, France; lmure_san@yahoo.com; 6 Department of Anatomy, “Iuliu Hatieganu” University of Medicine and Pharmacy, 400000 Cluj-Napoca, Romania; d.dumitrascu@yahoo.com; 7 Department of Medical Informatics and Biostatistics, “Iuliu Hatieganu” University of Medicine and Pharmacy, 400349 Cluj-Napoca, Romania; dleucuta@umfcluj.ro; 8 Department of Neurosciences, Discipline of Psychiatry and Pediatric Psychiatry, “Iuliu Hatieganu“ University of Medicine and Pharmacy, 400000 Cluj-Napoca, Romania; mihaelastanculete@yahoo.com

**Keywords:** antibiotic discovery, antibiotic development, automated antibiotic discovery, antibiotic resistance, computer-aided drug design, artificial intelligence, machine learning, deep learning, future of medicine

## Abstract

Over recent decades, a new antibiotic crisis has been unfolding due to a decreased research in this domain, a low return of investment for the companies that developed the drug, a lengthy and difficult research process, a low success rate for candidate molecules, an increased use of antibiotics in farms and an overall inappropriate use of antibiotics. This has led to a series of pathogens developing antibiotic resistance, which poses severe threats to public health systems while also driving up the costs of hospitalization and treatment. Moreover, without proper action and collaboration between academic and health institutions, a catastrophic trend might develop, with the possibility of returning to a pre-antibiotic era. Nevertheless, new emerging AI-based technologies have started to enter the field of antibiotic and drug development, offering a new perspective to an ever-growing problem. Cheaper and faster research can be achieved through algorithms that identify hit compounds, thereby further accelerating the development of new antibiotics, which represents a vital step in solving the current antibiotic crisis. The aim of this review is to provide an extended overview of the current artificial intelligence-based technologies that are used for antibiotic discovery, together with their technological and economic impact on the industrial sector.

## 1. Introduction

The average lifespan has extended by 23 years in the last century due to a number of factors, among which is the discovery of certain antibiotics, most notably Salvarsan in 1910 and Penicillin in 1928 [1].

After a short period of misleading triumph, scientists and physicians observed that micro-organisms were protected against the newly developed antibiotics by complex and numerous antibiotic resistance mechanisms. It is worth mentioning that in the 2nd, 3rd, 4th and 5th decades of the last century, the process of launching a new drug on the market was far less complicated than it is today, where it takes an average period of 12 years from the point of the drug discovery until market authorization [2,3,4].

Although bacterial resistance mechanisms existed before the discovery of synthetic antibiotics, the inappropriate use of antibiotics for medical, veterinary and agricultural purposes has further complicated the undesirable antibiotic resistance. Unfortunately, a progressive evolution of antibiotic resistance has led to the current antimicrobial crisis, which is responsible for 700,000 deaths per year worldwide [2]. Without a major breakthrough in antibiotic development, predictions show a number of 10 million deaths per year by 2050 [2]. The current epidemiologic studies have shown that the economic burden that is associated with antibiotic resistance is significant, and that each year in Europe and the United States these infections lead to 11 million additional hospitalization days and more than 20 billion dollars in additional social and healthcare costs [3,4,5,6].

If the major perspectives for the development of new antibiotics in the last two decades included resistance gene detection, genome sequencing and rapid pathogen determination [7,8,9], then artificial intelligence (AI), machine learning (ML) and neural networks (NN) have opened a new golden age of drug discovery and synthesis by processing enormous quantities of data almost instantaneously.

Initially, AI-based technologies were designed for simple and repetitive tasks and were considered limited in complex human-like processes, which use imagination and creativity. Nonetheless, sustained development in the field of AI technologies has greatly broadened and diversified their applications.

Traditional techniques of drug discovery are characterized by high costs, a prolonged period of synthesis, testing and implementation, expensive equipment, and extended human resources, which are probably the most difficult to obtain [2,3,4]. As an alternative, automated computer-aided drug-discovery techniques are considerably cheaper and faster, leading to a more rapid progression towards the pre-clinical and clinical testing phases.

The aim of this review is to provide an extended overview of the current artificial intelligence-based technologies that are used for antibiotic discovery.

## 2. Machine Learning and Deep Learning Technologies in Drug Development

Antibiotic-resistant bacteria represent a challenging and concerning aspect of modern medicine, with factors such as the decreased development of new antibiotics and the spread of multi-drug-resistant determinants aggravating the problem. However, the continuous development of artificial intelligence brings a new perspective to the field of antibiotic discovery.

The traditional experimental methods of discovering new antibiotics or improving existing ones are now being influenced by algorithms that were created by machine learning and neural networks, which allow larger in silico exploration and study. The main Artificial Intelligence technologies that are used in the analyzed studies are described in Table 1.

When it comes to machine learning, this type of technology consists of various computational methods that are based on previous experience [10,11,12]. The computers operate with so-called “raw data” in order to extract patterns and construct algorithms [10,11,12]. The efficiency and value of such an algorithm is closely correlated to the quality and sample size of the data that is used in the process [10,11,12]. Thus, it can be used in order to improve the performance of certain programs or to make predictions, while using the advantage of computational power and the ability to rapidly process large quantities of information [10,11,12].

Nevertheless, programs that are based on machine learning still require the attention of human specialists, in contrast to deep learning machines [10,11,12]. A computer that uses deep learning can understand complex phenomena by organizing hierarchies and splitting complex concepts into simpler ones, thereby continuously learning from itself [12].

Da Cunha et al. combined machine learning, spectroscopy and the antibiotic mechanisms of action and potency via high-throughput Fourier-transform infrared spectroscopy. This technique is based on the detection of certain metabolic fingerprints in order to assess the growth inhibition that is generated by the specific antibiotic, together with its mechanism of action. By analyzing specific antibiotics belonging to certain classes, it successfully predicted the mechanisms of action of different antibiotics belonging to the same class. Moreover, it was also capable of estimating antibiotic potency, which was measured by the metabolic fingerprints that were detected by Fourier-transform infrared spectroscopy, reflecting the cell alterations that were induced by the antibiotic [13].

Zoffman et al. also used machine learning by analyzing and searching through the Roche compound library, eliminating known antibiotics and other substances from other past antibiotic projects, prioritizing the remaining compounds based on novelty, potency, chemical structure, and the availability of purified powder material. These were further tested against four Gram-negative bacteria in order to assess their antibacterial activity. Moreover, the study aimed to show certain compound-induced phenotypic changes in relation to the lowest effective dose and the minimal inhibitory concentration, and to determine the mechanisms of action for novel compounds. Machine learning was used to determine and capture the specific bacterial phenotypic fingerprints in relation to certain mechanisms of action of different compounds, showing that compounds with the same mechanism of action induced similar phenotypic fingerprints. When it comes to novel compounds, these bacterial phenotypic fingerprints can be used to better establish the relationship between the structure and activity of certain antibacterial agents [14].

A deep learning approach towards novel antibiotic discovery was proposed by Stokes et al., by searching and formulating predictions using various databases [15]. After training and optimizing the model, it was used in order to identify potential antimicrobial molecules from the Drug Repurposing Hub [16]. This database consists of a large number of molecules that are being tested in various stages of research, in order to find new applications for them [16]. Finally, 99 molecules were identified and further empirically tested for antimicrobial inhibition; 51 of these compounds showed a strong inhibitory effect on a strain of *E. coli* [15]. During the clinical phase of investigation, the structural similarities of molecules from the training dataset and the predicted toxicity were also taken into consideration using a deep neural network in order to select the compounds with low structural similarity and the lowest toxicities [15]. The algorithm showed that halicin displayed strong growth inhibitory activity against *E. coli*, even on cells that persisted after treatment with ampicillin [15]. Being potent against multiple strains of antibiotic-resistant *E. coli*, the growth inhibitory potential of halicin was also tested on other pathogens, such as *M. tuberculosis*, carbapenem-resistant *Enterobacteriaceae* (CRE), *A. baumannii* and *P. aeruginosa*; it showed promising results, possessing strong inhibitory properties against CRE and *A. baumannii*, while also proving to be bactericidal against *M. tuberculosis*, but lacking efficiency against *P. aeruginosa* [15]. Halicin presents a complex and particular mechanism of action. It has been proven to sequester iron inside the bacteria, thereby disrupting its ability to maintain a normal electrochemical membrane gradient, thus inhibiting metabolism and resulting in cell death [15]. Additionally, halicin is a c-Jun N-terminal kinase (JNK) inhibitor [15].

Not only can machine learning be used in order to predict and discover novel antibiotics, but also to search a large amount of data, followed by the selection of certain compounds that meet the required criteria. Parvaiz et al. used machine learning in order to conduct a large search for compounds possessing the beta-lactamase inhibition quality. Of these 700,000 compounds, 74 were identified, after which they were subjected to empirical validation, revealing that eleven compounds were recognized as enhancers, while seven were inhibitors of CMY-10, which is a plasmid-encoded class C beta-lactamase. One compound presented great promise, being regarded as both a β-lactam enhancer and β-lactamase inhibitor. Moreover, machine learning facilitated the search for structurally similar compounds, after which 28 more were identified, all of them exhibiting β-lactamase inhibition potential and antibacterial activity [17].

Thus, the crossing between antimicrobial resistance and machine learning has allowed the development and improvement of numerous models that facilitate novel antibiotic discovery in order to make antimicrobial therapies more efficient. Moreover, new databases have been emerging, such as AntibioticDB, in order to aid the continuous research and development of known and new antibiotics [18]. Such kinds of databases consist of a large number of compounds in different stages of development, i.e., drugs under pre-clinical development, discontinued drugs or compounds in clinical trials. Thus, the ability of Artificial Intelligence to search and select certain compounds from these large databases, while taking into account various features, can greatly improve and speed up the process of drug development. Even though there are certain limitations to such databases, it is crucial that the challenges of antibiotic discovery and development be assessed through the cooperation between various institutions and entities.

With the arrival of the current global pandemic caused by severe acute respiratory syndrome coronavirus 2 (SARS-CoV-2) infection and with already more than five million deaths as a result, deep learning-based approaches are essential in identifying novel compounds to fight against the multiple variants of the virus and their mutations. While the traditional route for drug discovery may be too slow and expensive to keep up with the increasing transmissibility and the mutations of the virus, deep learning approaches can be essential in accelerating the drug-discovery process in this actual context. Studies have already used deep learning for drug repurposing, a technique which can assure the rapid identification of COVID-19 treatments. Choi et al., through a hybrid of deep learning- and molecular simulation-based screening procedures, identified azithromycin as a drug candidate for the targeting of RNA-dependent RNA polymerase, which was further confirmed to inhibit SARS-CoV-2 replication in vitro [19].

From another point of view, the COVID-19 pandemic comes with multiple secondary infections, many of them caused by multi-drug-resistant bacteria. Consequently, antimicrobial resistance becomes a more accelerated phenomenon, with additional negative consequences for the healthcare systems. Therefore, artificial intelligence methods, such as machine learning or deep learning, may help to achieve a faster delivery of promising antibiotic candidates [20].

Neither the empirical nor the modern technologies and approaches should be used exclusively, but rather a combination of these two, in order to assess the problem of antibiotic discovery in a coherent, comprehensive and efficient way.

## 3. Neural Networks and Antimicrobial Compounds

Inspired by the architecture and structure of the human brain, new AI technologies called neural networks have started to emerge. They consist of interconnected processing units and are based on pattern recognition technology. Moreover, the network learns from examples to perform certain tasks, even though it does not need a preset rule system, functioning instead through the constant adjustment of results in order to reach a target value [21].

Word embedding is a technique that is used in natural language processing in which words from a vocabulary are represented as vectors by using a significant number of words to form the pieces of the text as an input [22]. Bacteriocins are proteic or peptidic toxins that are produced by some bacteria in order to kill other bacteria or viruses that may endanger them, which represents one of the most promising perspectives on novel antibiotic discovery [23]. Among their mechanisms of action, pore-forming and permeabilization of bacteria, nuclease activity and DNA disruption, or inhibition of peptidoglycan formation are the most frequent [23].

Hamid et al. selected a word-embedding representation for each trigram from a protein sequence, and then used a Recurrent Neural Network (RNN), which is a subsequent type of artificial neural network, to distinguish between bacteriocin and non-bacteriocin sequences. The results showed that the novel technique can predict, with a statistically significant probability, six bacteriocins in Lactobacillus that were yet unknown, and the authors concluded that their RNN-based algorithm is the best automated method for the classification of bacteriocins compared to the current automated AI-based algorithms for biological sequence classification [22].

An artificial neural network (ANN) was used by Badura et al. to predict the antimicrobial properties and the biological and chemical effects of quaternary ammonium salts against *E. coli*. The study was based on the transformation of chemical information into three-dimensional models of imidazole chlorides and the generation of molecular descriptors via computational chemistry methods. The result was a high classification accuracy (95%, regression model: learning set R = 0.87, testing set R = 0.91, validation set R = 0.89), demonstrating that ANN-based systems can be successfully used to find efficient antimicrobial compounds [24].

## 4. Antimicrobial Peptides and Artificial Intelligence

The ever-increasing resistance toward conventional antibiotics has prompted the scientific community to broaden the area of research in order to find new ways to tackle emerging drug-resistant bacteria. This has led to antibacterial peptides receiving an increased amount of attention lately, and important research has been conducted in this field of interest, which shows great promise and frames antimicrobial peptides as new forms of anti-infectives, with some managing to kill the pathogens directly, while others intervene in modulating the immune response [25].

Fields et al. analyzed an automated system for peptide discovery and synthesis using ML and biophysical selection of minimal bacteriocin domains. The ML system was trained to design and test bacteriocin-derived peptides by using a sequence-free prediction algorithm. The protocol of the study provided 20 amino acid-peptide candidates (20-mers) for evaluation and in the initial phase the ML system generated a total of 28,895 20-mer peptides. In the next phase, sixteen sequences were selected for synthesis and then the antimicrobial, cytotoxicity, and hemolytic activities were further analyzed. The results demonstrated that bacteriocin-based peptides showed significant antimicrobial activity against *E. coli* and *P. aeruginosa,* and that the ML based method is an excellent approach for the discovery and synthesis of novel bacteriocin candidates [23].

Feng et al. explored the possibility of classification of antibacterial peptides using primary sequence information and Increment of Diversity with Quadratic Discriminant (IDQD) analysis [26]. IDQD represents a type of machine learning that aims to sequence pattern recognition [27]. While analyzing certain features of said sequences, it constructs a scheme in order to recognize and further predict patterns [27]. This type of technology has successfully been used in the analysis of the human genome [27]. The results showed an accuracy of 86.02%, a sensitivity of 74.31% and a specificity of 92.79% for identifying antimicrobial peptides. The authors concluded that their method is superior to all other automated methods that are currently used for antibacterial peptide classification [26].

The latest improvements in AI technologies provide better methods and give new perspectives to antimicrobial peptide identification, development and research. Bhadra et al. designed AmPEP, which is a model that analyzes the distribution pattern of amino acid properties using machine learning, i.e., a random forest algorithm [28]. One such algorithm uses multiple decision trees in order to reach a final result [27]. The algorithm groups amino acids into different categories based on their physicochemical properties [28]. In order to do so, the global percentage of amino acids of each class, the percentage of the frequency of transitions between two classes, and the distribution patterns of amino acids in each class are taken into consideration [28]. Thus, certain peptides have been considered and identified as antimicrobial peptides, which further boosts the research in this direction.

Immuno-informatics approaches have revolutionized the development of vaccines. In contrast to antibiotic use, vaccinations could help to prevent emerging infectious diseases, which would have a colossal impact on public health and antimicrobial resistance (AMR) [29].

Moreover, recent progress when it comes to different uses of antimicrobial peptides has also shown them as having great potential as vaccine adjuvants [30]. With their known role of modulating the immune response [25], the question was asked whether the peptides that influence the antigen-presenting cells could be predicted. Thus, Napgal et al. proposed several computer-aided prediction methods, which took into account the composition and position of epitopes, in order to be able to predict and even design immunomodulatory peptides [31].

Su et al. used a multi-scale convolutional network in order to identify and analyze antimicrobial peptides by training their model on four datasets and comparing it to other works, with significant results [32]. One such neural network can be used in order to achieve better performance when it comes to feature selection and potential fault identification [33]. This neural network model has two layers that encode each amino acid from the peptide sequence, then further analyzes and selects its features, outperforming most current state-of-the-art models when it comes to performance, while still needing improvements regarding the overall execution time [32].

Using hidden Markov models (HMMs), i.e., generative models that describe certain observational events that depend on intrinsic features, Fjell et al. managed to construct AMPer, which is a database that also functions as an automated discovery tool for antimicrobial peptides [34,35]. The algorithm enables the recognition of individual classes of antimicrobial peptides with remarkable accuracy [34]. Public sources were used, in addition to the Swiss-Prot database, in order to select the 1,045 mature peptides and 253 pro-peptides that make up the AMPer database [34]. These antimicrobial peptides consist of the major classes of antimicrobial peptides, such as defensins, cecropins, granulins and cathelicidins [34].

Cherkasov et al. used the predictive ability of an artificial neural net together with chemical descriptors in order to predict and design potent antimicrobial peptides based on the large databases of peptides. The study also aimed at proving that certain specific amino acid compositions and primary structures were needed for the peptide to possess antimicrobial activity. Using quantitative structure-activity relationship (QSAR), the team trained a model on two peptide databases, obtaining 100,000 peptides which were then sorted by hypothetical antimicrobial potential and grouped into four quartiles. The first two quartiles, consisting of the most promising candidates in terms of hypothetical antimicrobial activity, were further tested in vitro on a wide range of pathogens. Thus, 98% of the peptides belonging to the first quartile and 88% of the peptides from the second quartile were effective against *P. aeruginosa*. Moreover, two lead peptides were selected, due to their strong in vitro inhibitory effects, for in vitro testing against multiple highly resistant pathogens such as strains of multi-drug-resistant *P. aeruginosa*, methicillin-resistant *Staphylococcus aureus*, extended spectrum β-lactamase producing *Escherichia coli* and *Klebsiella pneumoniae*, and vancomycin-resistant *Enterococcus faecalis* and *Enterococcus faecium*. The two peptides demonstrated superior activity compared to other important antibiotics such as tobramycin, ciprofloxacin, ceftazidime and imipenem, which represent the most potent, highly utilized variants of their respective pharmacological classes. The study concluded that the two candidates HHC-10 and HHC-36, if appropriately formulated, could be highly effective against systemic infections, such as infections with *S. aureus* [36].

However, one downside of antimicrobial peptides is represented by their toxicity, with their hemolytic activity being the most notable one [37]. The two lead peptides that were identified in Cherkasov’s study have also shown minimal hemolytic activity at all concentrations, thus proving their pathogen-specific characteristics [36].

The development of machine learning has also made it possible for Cruz-Monteagudo et al. to develop a model capable of assessing both the potency and the toxicity of antimicrobial peptides. The combined use of machine learning and desirability theory allowed them to develop a multicriteria classification rule that managed to obtain a prediction accuracy of 80% [38].

Not only can antimicrobial peptides be discovered by using machine learning algorithms to analyze large quantities of data from various libraries, but recent studies have shown the potential of analyzing the toxins of various predators’ venom in order to reveal and test new antimicrobial peptides [39,40,41,42].

Grafskaia et al. performed a transcriptomic study of the sea anemone *Cnidopus japonicus* in order to extract, analyze and assess its peptides and their antimicrobial activity. Moreover, they developed an in silico machine learning search algorithm in order to discover toxin-like proteins, which further contained antimicrobial peptides, by taking into account the structural characteristics of amino acid sequences. With this technique, combined with transcriptomic data and proteomic profiling, ten peptides were selected and synthesized, out of which three (peptides A1, A3 and B1) exercised antimicrobial activity in the following manner: one was active on both Gram-positive and Gram-negative bacteria, while the other two only inhibited the growth of Gram-positive bacteria. The chemical structure of the peptide A1 consisted of an amino acid strand and alpha-helix that was similar to those of a toxin that can be found in another sea anemone *Stichodactyla helianthus* and is a potassium-channel inhibitor. The predicted structure of peptide A3 suggested more similarity to another peptide (A2) that exhibited anticancer properties. The assumed structure of peptide B3 suggested a similarity with GsMTx2, a toxin which inhibits mechanosensitive ion channels and is produced by the tarantula Grammostola spatulate. Even though the study did not manage to come up with many promising peptides that were capable of antimicrobial activity, it did show the great potential that these technologies have to discover, study, assess and test the antimicrobial activity of peptides that are found in the venom of various predators [39]. Antimicrobial peptides show great potential in targeting various strains of multi-drug-resistant bacteria, and the emergence and development of machine learning and other AI technologies can further boost research in this domain.

## 5. Specific Antibiotics for Specific Bacteria

Machine learning models are also useful in predicting more complex forms of antibiotic resistance in bacteria for which resistance has become a public health problem and for which the last-resort treatments have already been widely used. Macesic et al. used machine learning in order to predict phenotypic polymyxin resistance (PR) in Klebsiella pneumoniae clonal group 258. Their method used a reference-based approach that relied on variant calling and insertion sequence detection, and a reference-free approach that used the detection of k-mers. The reason for using ML models and this approach was based, on one hand, on the difficulties of applying other methods due to the incomplete identification of contributing PR mutations and the possible polygenic nature of PR. On the other hand, the increasing availability of bacterial whole-genome sequencing data permitted their exploration into the genotype-phenotype prediction of antimicrobial susceptibility testing. The best performance was obtained through the use of the reference-based approach and a curated input data set. The method can further be improved by conducting bacterial genome-wide association study (GWAS) filtering and by incorporating clinical data on antimicrobial exposure [43].

The outer membrane (OM) of the *Pseudomonas aeruginosa* bacterium is one of the most impenetrable barriers to antibiotics and still represents a challenge for drug permeability and drug discovery [44]. Mansbach et al. applied the machine learning algorithm “Hunting FOX” (“Hunting Fragments of X”), which is suitable for searching for fragments that match certain features, in order to construct a hybrid fragment-based design of molecules that are capable of permeating *Pseudomonas aeruginosa* [45,46]. The algorithm relied on traditional machine learning approaches, which are associated with natural language processing applications using n-grams and fragment-based drug design (FBDD) [45]. The algorithm considered all possible and relevant unique fragments within a set of compounds, in contrast to the other conventional FBDD which included only pre-defined small fragments from specific fragment libraries. The authors identified and validated a chemical vocabulary specific for Gram-negative bacterium *Pseudomonas aeruginosa* permeation, with a set of fragments that were expected to be responsible for the ability of antibiotics to permeate the OM of *Pseudomonas aeruginosa*, and obtained nine compounds that were expected to show good OM permeation, five of which were experimentally validated. Furthermore, they identified the permeation mechanism for the two molecules containing the most reported sub-molecular fragments, using molecular dynamics simulations [45].

Machine learning techniques were used by Smith et al. to optimize dosing regimens when using antibiotic combinations. This refers to the meropenem and polymyxin B association for the treatment of carbapenem-resistant *Acinetobacter baumannii*, which is another instance of critical priority antimicrobial resistance for which polymyxins remain, at present, the last line of treatment. Besides performing a genetic algorithm (GA) that was capable of defining the optimal dosing regimen for the antibiotic combination, authors also supplemented the pharmacodynamics data on the meropenem and polymyxin B association against *A. baumannii* and developed mechanism-based models in order to better describe the intrinsic activity of the compounds and their efficacy, either when separate or taken together. The approach was unidirectional, with polymyxin B affecting meropenem, based on the literature models data. The study generated six optimized drug regimens of the antibiotic combinations, which were capable of improving the probability of achieving bacterial eradication in 50 to 90% of the simulated patients. However, in order to provide this level of efficacy, the study underlined that the combination would require the use of doses above the ones that had been approved and/or recommended by the guidelines. Of great importance is also the use of aggressive monitoring strategies with regard to patients’ serum concentrations in order to maintain the usefulness of this combination in some patients [47].

Even though fewer cases and only occasional outbreaks have been reported lately, *Yersinia* spp. still poses a great threat to humanity, as it has been in the past, due to its large reservoir and ability to spread among many types of mammals [48]. Moreover, recent research has shown that *Yersinia* spp. is also capable of antimicrobial resistance, and concerns over a new outbreak have started to rise [49]. Hu et al. proposed a combined approach to target the antimicrobial resistance of *Yersinia* spp., more specifically targeting the virulence factors through machine learning and multiple conformational virtual screening, in order to find inhibitors of *Yersinia* protein kinase A(YpkA). Protein kinase inhibitor design represents a challenge because of the high similarity and plasticity of the catalytic site. By combining a machine learning method and multiple conformational high-throughput docking, the authors were able to discover YpkA inhibitors. YpkA is an essential virulence determinant that is involved in host actin cytoskeletal rearrangements and in the inhibition of phagocytosis. After training the machine learning model, achieving an accuracy of 70% and combining the algorithm with virtual screening, a total of 45 compounds were selected to be empirically tested for inhibitory properties on *Yersinia* spp., out of which seven managed to completely inhibit the growth of *Yersinia* spp., thus proving the potential of machine learning for discovering new compounds with antimicrobial activity [49].

## 6. Economic Impact

Although the scientific community has made significant progress in recent years in order to address the threat of multi-drug-resistant bacteria, not enough has been done for us to assume that the danger of new emerging strains has passed.

One of the factors that led to the so-called “antibiotics crisis” is represented by the ever-increasing cost of developing a new drug. DiMasi et al. analyzed the research and development costs of 106 randomly selected new drugs, which were obtained from 10 pharmaceutical companies [50]. The study revealed that the average cost for an approved new compound was $2870 million dollars in 2013, including the research, development, and post-approval costs [50]. Moreover, the average probability rate of a new drug to enter the market was 11.83% [50]. Making matters worse, around 4,000 immuno-oncology agents are currently in development, in contrast to the development of antibacterial compounds, of which only about 30-40 are in the clinical trial phases, and most of those are derivatives of already-existing antimicrobial agents [51]. This is due to a number of factors, such as the low price of antibiotics compared to other more expensive treatments, their short-term use compared with chronic treatments, and the limitations on the use of antibiotics in order to prevent their abuse, which would, in turn, deepen the problem.

The continuous use of conventional antibiotics has made it possible for multiple-drug-resistant strains to emerge, further aggravating the problem and prompting the scientific community to take action. The Centers for Disease Control and Prevention (CDC) estimated that, in the US alone, approximately 2 million people were infected with bacteria that were resistant to antibiotics in 2013, causing at least 23,000 deaths per year as a result [52]. In 2019, the number soared to 2.9 million antibiotic-resistant bacterial infections in the US, with as many as 35,000 deaths per year [52]. Moreover, in 2017 alone, 223,900 cases of Clostridioides difficile infections were reported in the USA, resulting in the deaths of 12,800 people [52]. A study conducted by Nelson et al. analyzed the healthcare costs of antibiotic-resistant bacterial infections and estimated the cost of treating community and hospital-onset infections at more than $4.6 billion in 2017 [53].

A Strength, Weaknesses, Opportunities and Threats (SWOT) analysis by Miethke et al. offers an in-depth perspective on the development of novel antibiotic drugs, mentioning the emerging artificial intelligence technologies as opportunities and future solutions for their ability to make hit discovery more efficient and reliable [20]. The extensive use of antibiotics in animals and the development of multi-drug-resistant bacteria render the process of antibiotic development useless, while fueling a never-ending race for finding novel antibiotics as current antibiotics become obsolete. It is a threat to antibiotics efficacy and is the basis of the need for the development of novel antibiotics that are more quickly introduced into medical practice and that are more precise in terms of their mechanisms of action.

The latest improvements in AI technologies are seen as an opportunity to discover novel antibiotics, develop and further improve existing ones, and better handle the current antibiotic crisis, due to their increased efficiency during the “hit discovery” phase of drug development [20]. Current algorithms and machine learning programs make it possible for large databases to be scanned and analyzed in order to select the best suitable “hit compounds” that possess antimicrobial activity.

Artificial intelligence could have a great impact on drug development, especially during the first phases. It could significantly reduce both the duration needed to select the candidate molecules as well as the cost per molecule. The costs of the early stages of candidate discovery (screening and hit generation, hit-to-lead and lead optimization) are estimated to be around 5–10 million euros, even without taking into consideration the so-called “attrition-molecules” that are abandoned later in the development process [20]. Additionally, this would mean a faster, more feasible process of drug development that would limit the existing threats, such as the rising death toll per year due to antimicrobial resistance, and the high socio-economic cost over future decades due to the increased hospitalization and treatment of multi-drug-resistant bacteria [20,54]. The World Health Organization (WHO) underlined the importance of innovative antibiotics in the latest report on the antimicrobial pipeline, emphasizing the impact that antimicrobial resistance has on certain vulnerable groups, such as children [55,56]. Moreover, none of the drugs that assess bacterial infections that are currently in development are able to sufficiently address the most dangerous and resistant pathogens, as evidenced by 30% of the neonates with sepsis losing their lives due to antimicrobial resistance to the first line of antibiotics [56].

## 7. Conclusions

Continuous improvement of AI technologies has opened the way to new perspectives of drug development, providing the necessary tools to efficiently treat drug-resistant bacteria. Insufficient antimicrobial agents and increased drug resistance have led to the unfolding “antibiotic crisis.” Increased cooperation between academic institutions and drug developing industries is needed in order to overcome the challenges that are currently being faced by patients and healthcare workers alike. Innovative strategies that accelerate the process and lower the cost of drug development represent an achievable way in which AI technologies can positively impact the pharmacological and healthcare industries.

## Figures and Tables

**Table 1:** Main Artificial Intelligence technologies used in the included studies.

Reference	Characteristics	Outcomes	Technology
da Cunha et al., 2021	Detecting certain metabolic fingerprints through spectroscopy; using ML technology to analyze and further predict the mechanism of action and potency of different antibiotics	Successfully predicted the mechanism of action; accurate estimation of the antibiotic potency	ML + high-throughput Fourier-transform infrared spectroscopy
Zoffman et al., 2019	Searching, identifying, and predicting potency of compounds with a random forest model	Assess phenotypic changes and antibacterial potency; predicted the phenotypic changes in compounds with identical and different mechanism of action	ML-random forest model
Stokes et al., 2020	Using DL and NN to search databases and predict potential antimicrobial compounds, further empirically testing them	Successfully combined AI technologies and clinical investigation; halicin displayed strong antibacterial properties	DL + NN
Parvaiz et al., 2021	Using ML to search for and identify potential candidates possessing beta-lactamase inhibition quality	Identified 74 compounds, out of which one showed great promise and further used ML in order to search for compounds structurally similar, concluding that all of the 28 additionally returned results had antibacterial activity	ML-random forest model
Hamid et al., 2019	Used neural networks in order to distinguish between bacteriocin and con-bacteriocin sequences	The algorithm can successfully predict and classify bacteriocins based on their sequence	RNN
Fields et al., 2020	Used ML to design and test bacteriocin-derived compounds and further assess their antimicrobial activity	The study designed and empirically tested compounds returned by the ML algorithm, with significant results	ML
Badura et al., 2021	Used ANN to generate computational chemistry models and identify and classify compounds	Transformed chemical information into computational models used to further search and identify antimicrobial compounds	ANN
Feng et al., 2019	Used IDQD in order to analyze and search for patterns in certain sequences and predict further patterns in antibacterial peptides	The study used this type of ML to successfully identify antimicrobial agents based on certain features of the antibacterial peptides	ML-IDQD
Bhadra et al., 2018	Used ML to analyze the distribution pattern of amino acids in antibacterial peptides	The model grouped amino acids based on certain properties in different groups and further predicted and identified antimicrobial peptides	ML-random forest model
Napgal et al., 2018	Used ML to search, analyze and predict peptides based on certain features	Analyzed peptides capable of inducting response of the APCs and further used ML to predict such peptides based on their structure	ML
Su et al., 2019	Used NN trained on various datasets to achieve performance in feature selection and structure analysis	Analyzed the features and structure of amino acids and peptides in order to identify novel antimicrobial peptides	NN
Fjell et al., 2007	Used Hidden Markov models to construct an algorithm that enables recognition of individual classes of antimicrobial peptides	Constructed a database that functions as a discovery tool for antimicrobial peptides	NN-Hidden Markov models
Cherkasov et al., 2009	Used NN to search various databases and identify and further design antimicrobial peptides	Screened a large number of peptides and selected the most potent ones for in vitro testing, further concluding that two compounds exhibited strong antimicrobial effects	NN
Cruz-Monteagudo et al., 2011	Used ML to create and define classification rules for antimicrobial peptides	The study aimed to assess both the toxicity and potency of antimicrobial peptides	ML
Grafskaia et al., 2018	Used ML to create an algorithm for the identification of toxin-like that also acted as antimicrobial agents	The combined ML and proteomic technologies showed the potential of such research, even though the study returned a small number of candidate peptides	ML
Macesic et al., 2020	ML was used to assess and quantify both bacterial resistance and susceptibility to certain antibiotics	Used ML to predict phenotypic polymyxin resistance in Klebsiella pneumoniae and to assess antimicrobial susceptibility	ML
Mansbach et al., 2020	The study used ML to construct and design molecules capable of penetrating the membrane of *Pseudomonas aeruginosa*	The algorithm constructed and considered every possible fragment-based design, obtaining five compounds that were experimentally validated and showed good membrane penetration	ML-Hunting Fox Algorithm
Smith et al., 2020	The study used ML in combination with genetic algorithms to assess intrinsic activity and efficacy of compounds	The study aimed to optimize dosing regimens when using antibiotic combinations, particularly against *A. baumannii*, the algorithm returning six regimens capable of eradicating the bacteria; even though these were not empirically tested	ML
Hu et al., 2007	The study used ML and conventional methods to find new antimicrobial agents to counter the antimicrobial resistance of *Yersinia* spp.	The study combined ML and multiple conformational high-throughput docking in order to find YpkA inhibitors; the algorithm returned 7 compounds that were empirically tested and showed antimicrobial activity	ML

ML: Machine Learning; DL: Deep Learning; NN: Neural Networks; RNN: Recurrent Neural Networks; ANN: Artificial Neural Networks; IDQD: Increment of Diversity with Quadratic Discriminant.
